# A comprehensive assessment of Ghana’s energy security dimensions and environmental sustainability in a multi-indices analysis

**DOI:** 10.1038/s41598-025-05407-0

**Published:** 2025-12-13

**Authors:** Michael Ayine Alpha, Eric B. Yiadom, Albert Ahenkan

**Affiliations:** 1 University of Professional Studies, Accra, Ghana; 2 University of Ghana, Legon, Ghana

**Keywords:** Energy security, Environmental sustainability, Herfindahl-Hirschman index (HHI), Carbon intensity index (CI), Energy diversification, Climate-change adaptation, Climate-change impacts, Climate-change policy, Energy and society, Environmental economics, Sustainability

## Abstract

This study presents a comprehensive evaluation of Ghana’s energy security and environmental sustainability using a multi-indices approach. Focusing on five key indicators: the Herfindahl–Hirschman Index (HHI), Shannon–Wiener Index (H), Simpson Index (D), Adjusted Shannon–Wiener Neumann Index (SWNI), and Carbon Intensity Index (CI) are employed to assess the concentration, diversification, and dependency of Ghana’s energy sources alongside carbon emissions. The results indicate a highly concentrated energy portfolio, with petroleum products and biomass dominating the energy mix. The HHI score of 0.23 reflects moderate market concentration, while the Shannon–Wiener Index of 1.5 and Simpson Index of 4.3 highlight a relatively diverse but still imbalanced energy mix. The SWNI1 score of 0.37, accounting for political risk, reveals a vulnerability due to reliance on imported energy. Furthermore, the Carbon Intensity Index of 5.50 kt CO_2_ per ktoe of energy consumed (2022) underscores significant carbon emissions, necessitating a sustainable energy transition. These findings emphasize the need to enhance domestic energy production, diversify energy sources, and adopt low-carbon technologies to improve Ghana’s energy security and environmental resilience.

## Introduction

The history of human advancement and development has been inextricably linked with the availability and consumption of energy in various forms^1^. While energy remains a fundamental input for economic activity, its accessibility and reliability vary significantly across regions, leading to disparities in development and energy security concerns^2^. A number of studies have established a direct link between energy use, economic growth, and standard of living^3^. However, energy availability is not evenly distributed geographically and so the demand and supply of it cause tensions among nations, particularly in the face of geopolitical dynamics, supply chain vulnerabilities, and environmental sustainability challenges^4^.

Apart from the availability of energy, the production, storage, and supply of energy have serious financial, environmental, and economic implications for countries and if not well-managed result in adverse consequences on the livelihood of the people^5^. Access to reliable, affordable and clean sources of energy becomes a major factor in determining the well-being of countries. Ensuring a consistent supply of energy resources, which eliminates the threats of supply disruptions, price fluctuations, and geopolitical tensions is known as energy security^6^. It is, therefore, imperative for countries to ensure energy security, whether by domestic production or through imports. Thus, this requires a critical analysis of the energy profile, that is the demand and supply dynamics of countries.

Developing countries have legitimate development imperatives to alleviate poverty, enhance infrastructure, and improve living standards^7^. Since energy is a fundamental input for industrialization, transportation, and technological advancement, these nations will inevitably consume more energy to drive their development^8^. As they expand their manufacturing sectors, modernize agriculture, and build critical infrastructure, their energy demand will rise to support increased production and economic activities^8^.

Ghana, like other developing countries, has seen significant economic growth in recent years, with an average Gross Domestic Product (GDP) of about 5.92% which is above the world’s average of 3.00% for the same period^9^. As Ghana embarks on its development agenda, increase in energy consumption is critical in achieving growth. However, in the wake of climate change, energy transition is essential to mitigate greenhouse gas (GHG) emissions from fossil fuel combustion, which is one of the major contributors to rising temperatures on our planet^10^. Shifting to renewable energy sources like solar, wind, and hydropower enhances environmental sustainability by minimizing environmental degradation and extreme weather risks^11^. Ghana is not exempt from this transition wave. The country has made several commitments, both nationally and internationally, to mitigate climate change by diversifying its energy mix to include renewable energy. Ghana has demonstrated its commitments to address climate change by adopting comprehensive energy transition strategies. In September 2023, Ghana unveiled its energy transition and investment plan during the United Nations (UN) General Assembly, outlining a pathway to achieve net-zero energy-related carbon emissions by 2060 through the adoption of low-carbon energy solutions across key sectors^12^. Ghana also remains committed to the Paris Agreement by implementing its Nationally Determined Contributions (NDCs), aiming to reduce greenhouse gas emissions by 64 MtCO_2_e by 2030 through renewable energy expansion, energy efficiency, and climate-resilient policies^13^.

This shift, while necessary for GHG, comes with significant challenges and potential adverse consequences for existing energy systems^14^. Transitioning from fossil fuel-based energy to renewable sources requires substantial modifications in energy infrastructure, including the modernization of power grids, the expansion of energy storage capabilities, and the integration of intermittent renewable sources such as solar and wind^15^. Furthermore, geopolitical dynamics surrounding energy security may shift, as nations that were once energy importers could become self-sufficient through domestic renewable energy production, altering global energy markets and trade relationships. Countries that lack the necessary financial and technological resources for a smooth transition may experience energy access disparities, leading to increased energy poverty and insecurity. To navigate these challenges, it is imperative for Ghana to assess it energy security situations to ensure that energy systems remain resilient, secure, and equitable throughout the transition process.

## Literature review

Energy security and environmental sustainability are two interdependent pillars of modern energy policy^16^. This interdependency between energy security and environmental sustainability has attracted the attention of many researchers. For instance, For instance, several studies^17–24^ have explored the relationship between energy security and environmental sustainability and established that a relationship exist between the two. Whereas, Baumann analyzed the energy security framework of the European Union (EU) and found persistent strategic deficits, suggesting that a more comprehensive approach is necessary^25^. Similarly, Gill et al. examined renewable energy variability in IEA and non-IEA countries, emphasizing that energy diversification is crucial for long-term security^26^. Their findings align with Obadi and Korcek, who applied HHI to rank European energy security and found that Italy led in diversification and reducing vulnerability to supply shocks^27^.

In the African context, Alemzero et al. assessed 28 African countries using composite factor analysis and found that most nations suffer high import dependence and carbon emissions, signaling systemic energy insecurity^28^. Focusing on Ghana, Ahunu and Ackah identified institutional weaknesses in the natural gas sector, which discourage independent power producer investments, affecting long-term security^29^. Similarly, examined the impacts of petroleum pricing deregulation, highlighting potential economic hardships due to fuel and transport cost volatility^30^.

Comparative assessments further illustrate Ghana’s position. For instance, Nigeria, while being a major oil producer, shows higher import dependency for refined products and greater geopolitical risk exposure^31,32^. Côte d’Ivoire, on the other hand, has advanced in regional energy trade and hydropower integration^33,34^. Acquah et al., used the Composite Energy Security Index to rank Ghana 17th globally and 6th in Africa over a 20-year period, indicating moderate performance but significant room for improvement^35^. Meanwhile, Kipkoech et al., analyzed renewable energy policies and found that regulatory frameworks such as feed-in tariffs (FITs) and the Renewable Energy Act (2011) have fostered growth in renewable energy adoption, though barriers such as high technology costs and limited financing mechanisms persist^36^.

These studies collectively highlight the complex nature of Ghana’s energy security challenges, spanning policy gaps, economic risks, supply diversity, and environmental concerns, thereby, attracting research interests in energy security analysis, especially in Ghana and the African sub-region which have received modicum attention in the subject matter. This paper therefore, seeks to contribute to the energy security discussion of Ghana through a different lens. It adopts a more integrated approach, applying multi-index analysis to offer a holistic view of Ghana’s energy security and environmental sustainability performance.

## Methodology

The focus of this paper is to evaluate the energy security of Ghana through a comprehensive analysis of energy sources using the Herfindahl–Hirschman Index (HHI), Shannon–Wiener Index (H), Simpson Index (D), adjusted Shannon–Wiener Neumann Index (SWNI) and Carbon Intensity Index (CI). The goal is to gain a nuanced understanding of the energy security landscape in Ghana by considering both the concentration of energy sources, diversity and dependency. Statistical analysis and visualization were performed to effectively interpret and present the diversity metrics.

### Data source and reliability

The paper utilises secondary data on Ghana’s carbon emissions, energy production, imports, and consumption including sources such as fossil fuels (oil, natural gas), and renewables (solar, hydroelectric, biomass). The studies relied on Ghana Energy Commission for energy data and the world Bank Group for CO_2_ and political stability data for countries. While these sources are reputable, there are inherent limitations that must be acknowledged.

Firstly, there may be time lags in the data, as different sources may publish updates at varying intervals. World Bank data may not always reflect the most recent political and economic conditions. Secondly, discrepancies between data sources may arise due to variations in data collection methodologies. For example, different studies or institutions might define and measure energy use, emissions, or stability factors differently, leading to slight variations in reported figures. Finally, regarding the time period covered, the latest complete dataset available from Ghana’s Energy Commission on energy sources corresponds to 2022 as of 2023 and that for CO_2_ from the World Bank was 2020 and for political stability factor was 2021.

### The Herfindahl–Hirschman index (HHI)

The HHI is a standard metric used to measure market concentration in various industries, including energy. In the context of energy security, HHI is employed to assess the level of reliance on a limited number of energy sources. Analysing the concentration of Ghana’s energy sources using HHI helps policymakers identify risks associated with over-reliance on specific energy imports. HHI has previously been applied in energy security research. For instance, The European Commission and U.S. Energy Information Administration frequently use HHI to assess electricity and fuel market competition^37,38^. Obadi & Korcek^27^ and Acquah et al.^35^ applied HHI in their studies. The HHI is represented as:

1 $$HHI = \mathop \sum \limits_{{i = 1}}^{s} xi^{2}$$

Where *x*i is the market share. ∑ denotes adding the squared market shares.

### The Shannon–Wiener index (H)

The H index originally developed for biodiversity studies, has been adapted for energy security research to measure the diversity of energy sources. Given Ghana’s dependence on hydroelectricity, petroleum and natural gas, this index helps quantify the level of energy diversification and identify areas needing improvement. Researchers have taken interest in the H index for analysing energy security. For instance, Ranjan and Hughes^39^ applied the H index to analyze energy security in India, while Alemzero et al.,^28^ used the H index to assess energy security in 28 countries in Africa. The H index formula is represented as:

2 $$H= - \mathop \sum \limits_{{i=1}}^{s} {\text{P}}i*{\text{~In}}\left( {{\text{P}}i} \right)$$

Where: Pi is the proportion of each individual energy source, In is the natural log function, the negative sign ensures that H remains positive, as entropy increases with diversity. **∑** denotes adding the proportion of each individual energy source.

### The Simpson index (D)

The Simpson index is very similar to the H index but is a dominant index that provides an alternative way of measuring dependence and diversity. Unlike the H index, which gives equal weight to all sources, the Simpson index emphasizes dominant sources, making it ideal for identifying over-reliance on a single energy type. It provides an opportunity to confirm or contradict the H index. It is particularly useful for assessing Ghana’s dependence on a few major energy sources (e.g., petroleum and hydro).

The Simpson index is represented as:

3 $$D=~\frac{1}{{\mathop \sum \nolimits_{{i=1}}^{n} P{i^2}}}$$

*Pi* is the proportion of each individual energy source. ∑ denotes adding the squared proportion of each individual energy source.

### The adjusted Shannon–Wiener neumann index (SWNI)

The SWNI allows for other indicators or variables to be added to the Shannon–Wiener index model. It improves upon the original H Index by incorporating external risk factors such as political stability and indigenous production. Ghana imports a significant portion of its petroleum and natural gas from politically unstable regions such as Nigeria. SWNI helps quantify the risks associated with these imports. Munoz et al., (2015) applied a similar adjusted index to assess political risks in global energy markets^40^. For example, if the political stability factor alone is included, the index takes the form:

4 $$SWN{I_1}= - \mathop \sum \limits_{{i=1}}^{s} {\text{b}}i*{\text{P}}i*{\text{In}}\left( {{\text{P}}i} \right)$$

Where b*i* is the political stability factor of the country from where imports are coming. The (‘bi’) used in this study is derived from the World Bank’s Worldwide Governance Indicators (Political Stability and Absence of Violence/Terrorism).

To include the share of indigenous production, the SWNI index can be modified as follows:

5 $$SWN{I_2}= - \mathop \sum \limits_{{i=1}}^{s} {\text{b}}i*{\text{P}}i*~\left[ {{\text{In}}\left( {{\text{P}}i} \right)\left( {1+{\text{g}}i} \right)} \right]$$

where gi represents the indigenous production.

### Carbon intensity index (CI)

The CI quantifies the amount of CO_2_ emissions produced per unit of energy generated or consumed. It is a critical tool for assessing the environmental impact of energy production and consumption. A lower carbon intensity indicates a cleaner and more environmentally friendly energy system. As Ghana aims to transition toward low-carbon energy sources to enhance environmental sustainability, the CI provides insights into how different energy sources contribute to emissions. The CI is widely used in analysing energy systems and carbon emissions. For instance, Jayachandran et al.^41^ analysed carbon intensity in developing countries using CI. The CO_2_ data used here does not account for indirect emissions such as those embodied in imported petroleum products. It also excludes emissions from Land Use, Land Use Change and Forestry (LULUCF).

The CI model is represented as:

6 $$CI=\frac{{\mathop \sum \nolimits_{{i=1}}^{s} {\text{C}}{{\text{O}}_2}}}{{\mathop \sum \nolimits_{{i=1}}^{s} x }}$$

Where CO_2_ is kiloton of Carbon Dioxide equivalent. *x* is ktoe of final energy consumed. ∑ denotes adding the CO_2_ and energy consumption from different time periods.

## Results

The results of the energy security analysis are as follows: “Energy portfolio and concentration assessment”, “Assessment of the market structure of the power sector”, “Diversity and dependence assessment with the Shannon–Wiener index”, “Diversity and dependence assessment with the Simpson Index”, “Assessment of political risk”, “Assessment of indigenous production: adjusted Shannon–Wiener Newman index (SWNI_2_)”, and “Assessment of CO_2_ Intensity Index” for each set of results, analysis and discussion focus on the implication of low or high computed indices and their implications to the energy security of Ghana.

### Energy portfolio and concentration assessment

The HHI helps determine the market concentration levels of various energy supplies^42^. We applied the HHI_1_ to analyse the level of concentration for the entire industry by energy source. This involves quantifying the relative share of each energy source in the total energy mix and then calculating the HHI_1_ to understand whether certain sources dominate the market or if there is a balanced distribution. This approach is to minimise any challenges that could confront the study due to the many suppliers in the entire sector, with petroleum products alone having over 25 suppliers and suppliers of biomass is very difficult to determine^43^. The HHI analysis by energy source are shown in Table 1.

$$HH{I_1}=\mathop \sum \limits_{{i=1}}^{s} x{i^2}$$

**Table 1:** Energy market concentration. Source: Authors computations.

Energy supplied 2022	ktoe (x)	Proportion ktoe (*n*/*N*)	*xi*	*xi* ^2^
Crude oil	4147	4147/16,648	0.2490	0.0620
Natural gas	3472	3472/16,648	0.2085	0.0434
Hydro	704	704/16,648	0.0422	0.0017
Solar	14	14/16,648	0.0008	0.0000
Biomass	3993	3993/16,648	0.2398	0.0575
Petroleum products	4318	4318/16,648	0.2593	0.0672

Note that about 98% of petroleum products are imported and, therefore, need to be added so as to account for all energy supplied within the year and not just primary energy supplied. Thermal-generated electricity is accounted for in crude oil, natural gas, and petroleum products.

**s** (number of energy sources) = 6.

n (Individual energy source).

**N** (total ktoe of energy supplied) = 16,648.

*x*i is the market share.

*x*i^2^ is the square of the market share.

$${\text{\varvec{\Sigma}~}}\left( {{\text{sum}}} \right)~of~x{i^2}=0.23$$

$${\mathbf{HH}}{{\mathbf{I}}_1}=0.06+0.04+0.00+0.00+0.06+0.07=0.23$$

**Fig. 1:**
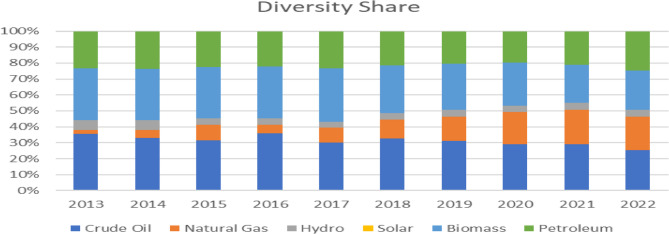
Energy source concentration levels and diversity. Source: Authors computations

Analyzing the energy source concentration levels over the last ten years for a variety of energy sources provides valuable insights into the nation’s energy landscape, sustainability efforts, economic performance, and commitments to achieving a favourable HH_1_. Note that about 98% of petroleum products are imported and, need to be added so as to account for all energy supplied within the year and not just primary energy supplied. Thermal-generated electricity is accounted for in crude oil, natural gas, and petroleum products. Specifically, we have explained how different levels of HHI_1_ indicate varying degrees of market concentration and how this affects energy security. The different values of *x*i in the table above indicate the energy mix of Ghana in 2022. The HHI_1_ value of 0.23 suggests moderate concentration, meaning that while the country has some level of diversification, it remains vulnerable to supply disruptions due to its reliance on a few key energy sources, particularly petroleum and natural gas. See Fig. 1 below.

**Fig. 2:**
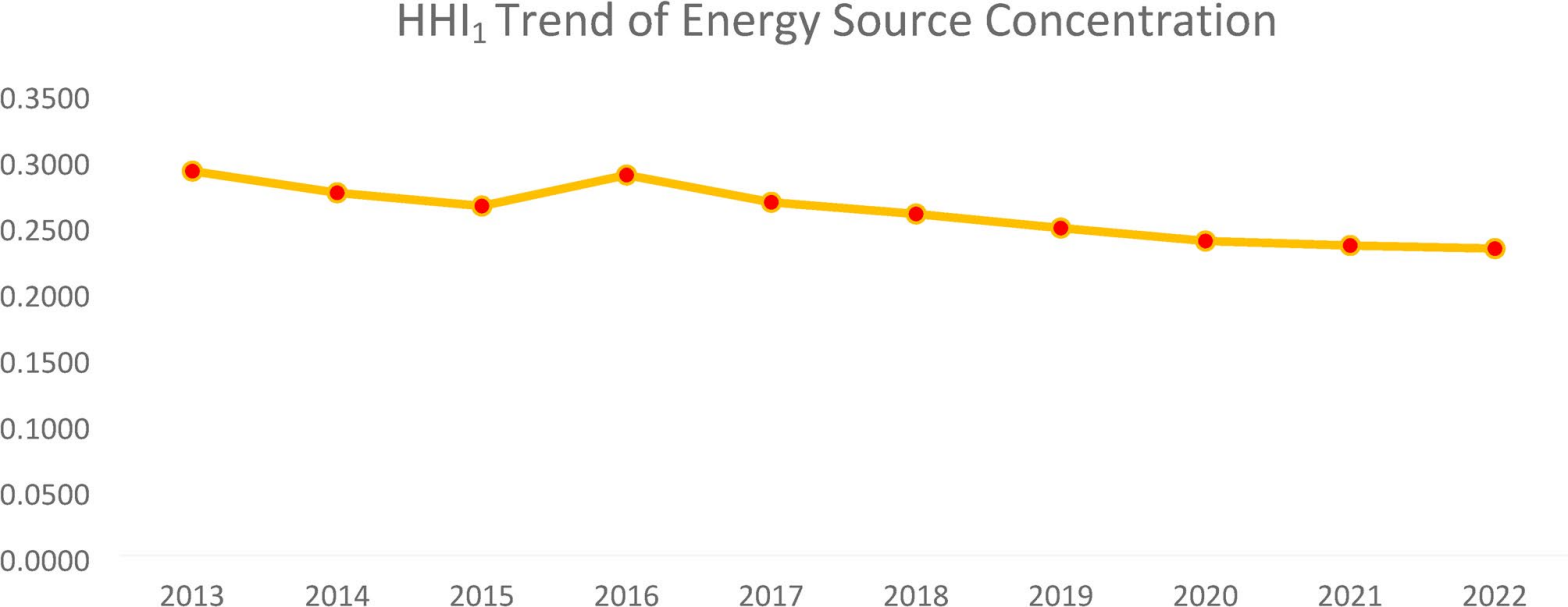
Energy source concentration. Source: Authors computations

By tracking the HHI_1_ of Ghana’s energy sources over a period of ten years, we can make indirect inferences about the changes in energy diversity within that country’s energy sector. See Fig. 2 below.

The HHI shows a general decline over the years, suggesting that Ghana’s energy mix has become more diversified over time. Between 2013 and 2015, the HHI slightly declined, but there was a brief increase in 2016, indicating a temporary rise in concentration. The decline in HHI implies Ghana is reducing dependence on a few dominant energy sources, which can enhance energy security and sustainability. The diversification trend could be due to increased integration of renewable energy sources, such as solar and wind, alongside traditional fossil fuel and hydroelectric power. Policymakers should continue to promote investments in varied energy sources to further strengthen resilience against supply shocks.

### Assessment of market structure of the power sector

By identifying suppliers of the power sector and obtaining market share data, the structure of the market could be determined by HHI_2_. The HHI_2_ can be used to assess the degree of concentration among suppliers within the power sector. Table 2 shows how it can be applied to assess suppliers in the power sector:

7 $${\text{HH}}{{\text{I}}_2}=\mathop \sum \limits_{{i=1}}^{s} x{i^2}$$

**Table 2:** Power sector generation mix. Source: Authors computations.

Energy suppliers 2022	ktoe (x)	Proportion ktoe ($\frac{n}{N}$)	xi	xi^2^
Akosombo	677.90	677.90/3647.83	0.1858	0.0345
Kpong	105.45	105.45/3647.83	0.0289	0.0008
Bui	248.56	248.56/3647.83	0.0681	0.0046
Takoradi Power Company (TAPCO)	237.26	237.26/3647.83	0.0650	0.0042
Takoradi International Company (TICO)	248.56	248.56/3647.83	0.0681	0.0046
Tema Thermal 1&2 Power Plant	128.04	128.04/3647.83	0.0351	0.0012
Kpone Thermal Power Plant	150.64	150.64/3647.83	0.0413	0.0017
Ameri Plant	173.240	173.240/3647.83	0.0475	0.0023
Sunon Asogli Power (Ghana) Ltd	399.2	399.2/3647.83	0.1094	0.0120
Karpowership	338.95	338.95/3647.83	0.0929	0.0086
Amandi (Twin City)	151.39	151.39/3647.83	0.0415	0.0017
AKSA	248.56	248.56/3647.83	0.0681	0.0046
Cenpower	256.09	256.09/3647.83	0.0702	0.0049
Others	283.99	283.99/3647.83	0.0779	0.0061

**s** (number of energy sources) = 14.

*x* (Individual energy source).

**N** (total dependable power capacity) = 3647.

*x*i is the market share.

*x*i^2^ is the square of the market share.

$${\text{\varvec{\Sigma}~}}\left( {{\text{sum}}} \right){\text{~of}}~x{i^2}=0.09$$

$${\mathbf{HHI}}_{2} = 0.04 + 0.00 + 0.01 + 0.00 + 0.01 + 0.00 + 0.00 + 0.00 + 0.01 + 0.01 + 0.00 + 0.01 + 0.01 + 0.01$$

$${\mathbf{HH}}{{\mathbf{I}}_2}=0.09$$

**Fig. 3:**
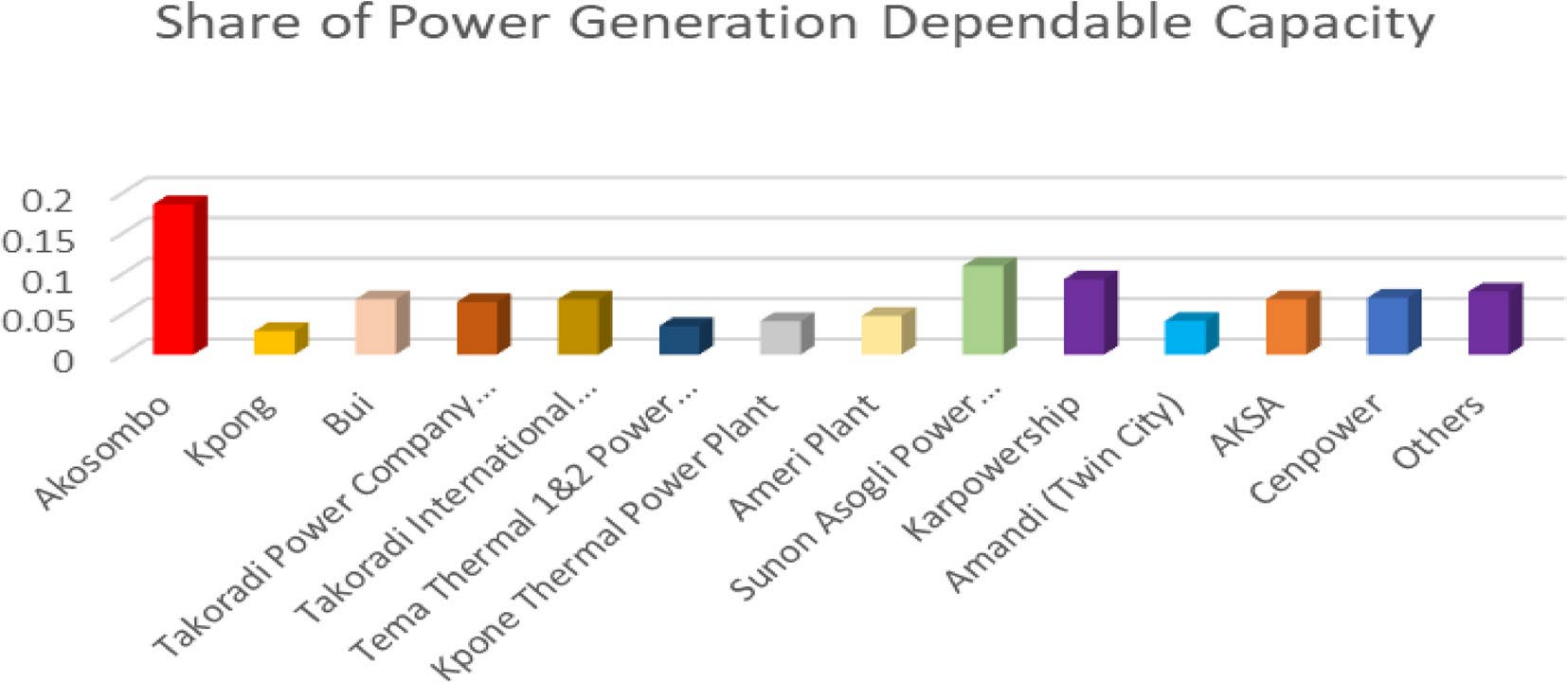
Power sector market share. Source: Authors computations

See Fig. 3 below for producer market share of the power sector.

Figure 3 above presents the share of power generation dependable capacity in Ghana, showcasing contributions from various power plants and energy sources. The Akosombo Hydroelectric Dam has the highest share of dependable capacity, indicating that hydroelectric power remains a critical component of Ghana’s electricity generation. The presence of several thermal plants, including Takoradi Power Company, Kpone Thermal Power Plant, and Sunon Asogli Power Plant, suggests a mix of gas-fired and oil-based thermal power stations. Also, significant contribution form Independent Power Producers (IPPs): Karpowership, Amandi, AKSA, and Cenpower are among the notable IPPs contributing to Ghana’s electricity generation, highlighting the importance of private sector participation. The data suggests that while hydro power is still a significant contributor, thermal and IPP contributions are ensuring a diversified and more resilient energy mix. However, renewable energy sources (solar, wind) is conspicuously missing in the Ghana’s power generation mix.

### Assessment of diversity and dependence with Shannon–Wiener index (H)

The Shannon–Wiener Index, also called the Shannon Diversity Index is widely used in ecology studies to measure the diversity of species within a biological community^44^. It can also be applied to assess energy diversity and dependence in the context of the energy sector^39^. This index provides a way to understand how different energy sources contribute to the overall energy mix and whether the energy supply is reliant on a few dominant sources or is more diversified as presented in Table 3.

8 $${\text{H}}= - \mathop \sum \limits_{{{\text{i}}=1}}^{{\text{s}}} {\text{P}}i*~{\text{In}}\left( {{\text{P}}i} \right)$$

**Table 3:** Shannon–Wiener index energy diversity and dependence. Source: Authors computations.

Energy supplied 2022	Ktoe (*n*)	Proportion (*n*/*N*)	Pi	ln pi	Pi ln Pi
Crude Oil	4147	4147/16,648	0.2490	− 1.3899	− 0.3462
Natural Gas	3472	3472/16,648	0.2085	− 1.5675	− 0.3269
Hydro	704	704/16,648	0.0422	− 3.1632	− 0.1337
Solar	14	14/16,648	0.0008	− 7.0809	− 0.0059
Biomass	3993	3993/16,648	0.2398	− 1.4277	− 0.3424
Petroleum Products	4318	4318/16,648	0.2593	− 1.3494	− 0.3500

s (number of energy sources) = 6.

n (Individual energy source).

*Pi* is the proportion of each individual energy source.

*In* is the natural log.

N (total ktoe of energy supplied for 2022) = 16,648.

$${\text{\varvec{\Sigma}~}}\left( {{\text{sum}}} \right){\text{~of~Pi~Inpi}}= - 1.51$$

$$\begin{aligned} H &:= - (0.25 \times In\left( {0.25} \right)+0.21 \times In\left( {0.21} \right)+0.04 \times In\left( {0.04} \right)+0.00 \hfill \\ & \quad \times In\left( {0.00} \right)+0.24 \times In\left( {0.24} \right)+0.26 \times In\left( {0.26} \right) \hfill \\ \end{aligned}$$

$$\begin{aligned} H &:= - \bigg(0.25 \times \left( { - 0.39} \right)+0.21 \times \left( { - 1.57} \right)+0.04 \times \left( { - 1.36} \right)+0.00 \hfill \\ & \quad \times \left( { - 7.08} \right)+0.24 \times \left( { - 1.43} \right)+0.26 \times \left( { - 1.35} \right)\bigg) \hfill \\ \end{aligned}$$

$$H:= - \left( { - 0.35+ - 0.33 + - 0.13 + - 0.01 + - 0.34 + - 0.35} \right)$$

$$H=1.51$$

**Fig. 4:**
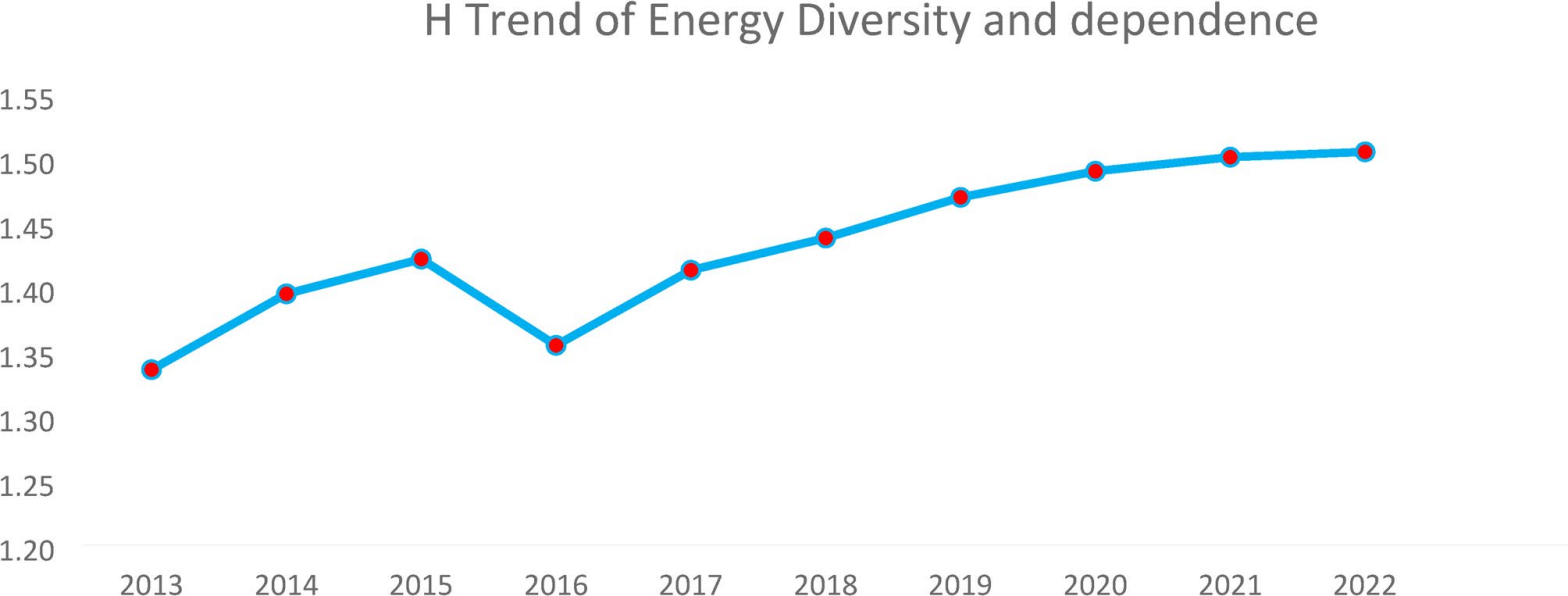
Shannon–Wiener Index trend of energy diversity and dependence. Source: Authors computations

Figure 4 above presents the H index trend for Ghana’s energy sources, reflecting changes in energy diversity and dependence from 2013 to 2022. The index increased from 2013 to 2015, then dipped in 2016, suggesting a temporary reduction in diversification possibly due to increased reliance on a particular energy source. The general upward trend in H suggests that Ghana’s energy mix has become progressively more diverse over the years. The increasing H index suggests Ghana is reducing dependence on a few dominant energy sources, enhancing energy security and resilience.

### Assessment of diversity and dependence with the Simpson index (D)

The Simpson gives more weight to common or dominant species. In this case, a few rare energy sources will not affect the diversity as with the Shannon–Wiener Index model^45^. The strength of the Simpson index is that the model presents different approaches to suit the study environment. In this context, we applied the inverse Simpson Index also known as Simpson’s Reciprocal Index and present the results in Table 4:

9 $${\text{D~}}= \frac{1}{{\begin{array}{*{20}{c}} {~~~} \\ {\mathop \sum \nolimits_{{{\text{i}}=1}}^{{\text{n}}} {\text{P}}{i^2}} \end{array}}}$$

**Table 4:** Simpson index energy diversity and dependence. Source: Authors computations.

Energy supplied 2022	Ktoe (*n*)	Proportion (*n*/*N*)	Pi	Pi^2^
Crude oil	4147	4147/16,648	0.2490	0.0620
Natural gas	3472	3472/16,648	0.2085	0.0434
Hydro	704	704/16,648	0.0422	0.0017
Solar	14	14/16,648	0.0008	0.0000
Biomass	3993	3993/16,648	0.2398	0.0575
Petroleum products	4318	4318/16,648	0.2593	0.0672

**s** (number of energy sources) = 6.

n (Individual energy source).

**N** (total ktoe of energy supplied) = 16,648.

*Pi* is the proportion of each individual energy source.

*Pi*^2^ is the squire of the proportion of each individual energy source.

$${\text{\varvec{\Sigma}~}}\left( {{\text{sum}}} \right)~of~p{i^2}=00.6+0.04+0.00+0.00+0.06+0.07=0.23$$

10 $${\mathbf{D}}=\frac{1}{{0.23}}=4.35$$

**Fig. 5:**
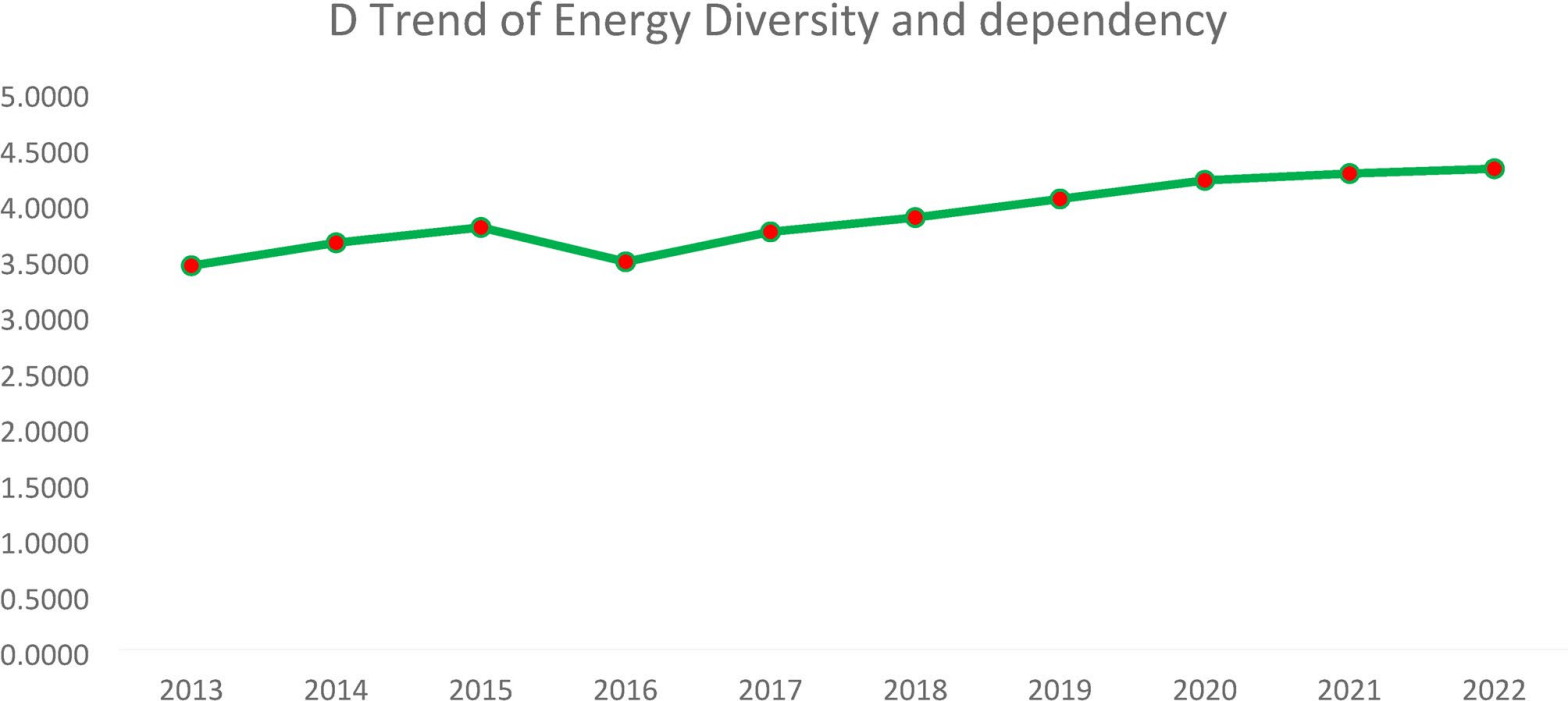
Simson index trend of energy diversity and dependency. Source: Authors computations

A trend analysis of the Simpson Index over a decade for the energy diversity provides valuable insights into the country’s energy sector’s development with regards to the efforts and commitments towards ensuring well diversified energy mix. See Fig. 5.

Figure 5 above provides the trend of the Simpson Index for energy source diversity in Ghana from 2013 to 2022. The Simpson Index, a measure of diversity, shows a general upward trend over the decade, indicating increasing diversification of energy sources. There are minor fluctuations, particularly a dip around 2016, but the overall trajectory suggests a steady improvement in energy mix diversity. The increasing values imply a more balanced reliance on different energy sources, which can enhance energy security and sustainability. The trend stabilizes around 2021–2022, suggesting Ghana has reached a relatively stable level of energy diversity.

### Assessment of political risk with adjusted Shannon–Wiener Newman index (SWNI_1_)

The SWNI_1_ removes the limitations of the H index. The SWNI_1_ allows for other indicators to be added to the Shannon–Wiener index model. We assess the political risk of importing fuels for Ghana based on import data for 2022 by incorporating political stability factor^46^. Even though Ghana is a net exporter of crude oil it still imports crude from the United States for the refineries^47^. Ghana produces significant natural gas but compliments domestic production with Nigerian gas through the West African Gas Pipeline^48^. Another energy that is heavenly imported is petroleum products. The major supplier of Ghana’s Petroleum products is the British Petroleum Pls from its USA subsidiary^43^. The SWNI_1_ will consider political risk from Nigeria, and The United States of America which are the origin of Ghana’s energy imports. Electricity imports are included from the analysis paint a holistic picture despite the low import figures which are not significant enough to impact Ghana’s energy security. Electricity imports mostly Ivory Coast for the past few years have decreased drastically with the 2022 figure at 37 GWh representing about 0.1% of electricity consumption for 2022^48^. We infused the political stability factor from to module to observe the impact on the H index^49^. For example, if the political stability factor alone is included, the index takes the form:

11 $$SWN{I_1}= - \mathop \sum \limits_{{i=1}}^{s} {\text{b}}i*{\text{P}}i*{\text{In}}\left( {{\text{P}}i} \right)$$

Equation 11 is computed and presented in Table 5.

**Table 5:** Political risk of imports. Source: Authors computations.

Energy imports 2022	Ktoe (*n*)	Proportion (*n*/*N*)	Pi	ln (pi)	bi	bi Pi	biPi ln Pi
Crude oil	31.64	31.64/4858	0.0065	− 5.0340	0.0049	0.000	− 0.0002
Natural gas	501.9	501.9/4858	0.1033	− 2.2700	− 1.779	− 0.184	0.4173
Petroleum	4322	4322/4858	0.8895	− 0.11706	0.5421	0.482	− 0.0565
Electricity	3.18	3.18/4858	0.0006	− 7.3315	− 0.9528	− 0.006	0.0046

**s** (number of energy sources) = 4.

n (Individual energy source).

**N** (total ktoe of energy imported) = 4858.

*Pi* is the proportion of each individual energy source.

*In* is the natural log.

bi is the political stability factor of the country from where imports are coming.

$${\text{\varvec{\Sigma}~}}\left( {{\text{sum}}} \right)~of~bi*pi*In\left( {pi} \right)=0.48$$

$${\mathbf{SWN}}{{\mathbf{I}}_1}~= - 0.00~+~0.42+ - 0.06~+~0.01$$

$${\mathbf{SWN}}{{\mathbf{I}}_1}~= 0.37$$

### Assessment of Indigenous production: adjusted Shannon–Wiener Newman index (SWNI_2_)

Domestic energy production plays a crucial role in enhancing a country’s energy security by reducing its reliance on imported energy sources. More especially for countries that import from unstable regions. Incorporating indigenous energy production into the ASWN index analysis provides a holistic view of a country’s energy security, considering not only the availability and reliability of energy sources but also the strategic, economic, and environmental implications of domestic energy production: To include the share of indigenous production, the SWNI index is modified in Eq. (11) and the results from the estimation is shown in Table 6.

12 $$SWN{I_2}= - \mathop \sum \limits_{{i=1}}^{s} {\text{b}}i*{\text{P}}i*\left[ {{\text{In}}\left( {{\text{P}}i} \right)\left( {1+{\text{g}}i} \right)} \right]~$$

**Table 6:** Impact of domestic production. Source: Authors computations.

Energy produced 2022	Ktoe (*n*)	Proportion (*n*/*N*)	Pi	ln (pi)	bi	bi Pi	biPi lnPi	gi	1 + gi	biPi In (Pi) (1 +gi)
Crude oil	7246	7246/1239	0.58	− 0.2718	0.0049	0.0029	− 0.0016	0.42	1.42	− 0.0022
Natural gas	2973	2973/1239	0.24	0.0000	− 1.779	− 0.427	0.6093	0.17	1.17	0.7144
Petroleum	180	180/1239	0.01	− 0.11706	0.5421	0.0079	− 0.0333	0.12	1.01	− 0.0337
Electricity	1992	1992/1239	0.16	− 1.7791	− 0.9528	− 0.153	0.2799	0.01	1.12	0.3123

**s** (number of energy sources) = 4.

n (Individual energy source).

**N** (total ktoe of domestic energy production) = 1239.

*Pi* is the proportion of each individual energy source.

*In* is the natural log.

*bi* is the political stability factor of the country from where imports are coming.

*gi* represents the indigenous production.

$$\begin{aligned} & {\text{Domestic~Crude~Oil~Imports}}:{\text{~Pi}}=0.58,~gi~=~0.42,~In\left( {Pi} \right)= - 0.27,~bi*Pi~=0.00,~ \hfill \\ & \quad bi*Pi*~\left( {InPi} \right)=~ - 0.00,~bi*Pi*\left[ {In\left( {Pi} \right)\left( {1+gi} \right)} \right]=~ - 0.00 \hfill \\ \end{aligned}$$

$$\begin{aligned} & {\text{Domestic~Natural~Gas~Imports}}:{\text{~Pi~}}=0.24,~gi~=~0.17,~In\left( {Pi} \right)=~0.00,~ \hfill \\ & \quad bi*Pi~=~ - 0.43,~bi*Pi*\left( {InPi} \right)=~0.61,~bi*Pi*~\left[ {In\left( {Pi} \right)\left( {1+gi} \right)} \right]=~0.71 \hfill \\ \end{aligned}$$

$$\begin{aligned} & {\text{Domestic~Petroleum~Imports}}:{\text{~Pi~}}=0.01,~gi~=~0.12,~In\left( {Pi} \right)=~ - 0.12,~bi*Pi~=~0.00, \hfill \\ & \quad ~bi*Pi*\left( {InPi} \right)=~ - 0.03,~bi*Pi*\left[ {In\left( {Pi} \right)\left( {1+gi} \right)} \right]=~ - 0.03 \hfill \\ \end{aligned}$$

$$\begin{aligned} & {\text{Domestic~Electricity~Imports}}:{\text{~Pi}}=0.16,~gi~=~0.01,~In\left( {Pi~} \right)=~ - 1.78,~bi*Pi~=~ - 0.15,~ \hfill \\ & \quad bi*Pi*\left( {InPi} \right)=~0.28,~bi*Pi*~\left[ {In\left( {Pi} \right)\left( {1+gi} \right)} \right]=~0.31 \hfill \\ \end{aligned}$$

$${\text{\varvec{\Sigma}~}}\left( {{\text{sum}}} \right){\text{of~b}}i*{\text{P}}i*~\left[ {In\left( {Pi} \right)\left( {1+gi} \right)} \right]= 1.82$$

$${\mathbf{SWN}}{{\mathbf{I}}_2} = - 0.00 + 0.71 + - 0.03+ 0.31$$

$${\mathbf{SWN}}{{\mathbf{I}}_2} =0.98$$

**Fig. 6:**
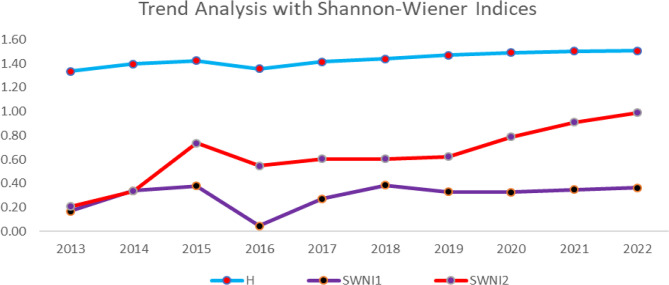
Trend of political risk and domestic production. Source: Authors computations

Figure 6 presents a ten-year trend analysis of the Shannon–Wiener Index and Adjusted Shannon–Wiener Index to assess how political risk and domestic production affects the original Shannon–Wiener Index.

Figure 6 above illustrates a 10-year trend analysis of the H index alongside its adjusted versions; SWNI_1_ (incorporating political risk) and SWNI_2_ (accounting for domestic production). The overall H index remains relatively stable, with a slight upward trend, suggesting a steady level of energy source diversity. SWNI_1_, which factors in political risk, exhibits fluctuations, notably a sharp decline around 2016, followed by gradual recovery. This suggests that political instability had a temporary negative impact on energy diversity. SWNI_2_, reflecting domestic production influence, shows a more pronounced upward trend, particularly after 2016. This indicates that increasing domestic energy production has contributed positively to energy diversity over time. The divergence between the indices highlights the significant impact of political and domestic production factors on energy source diversity in Ghana.

### Assessing CO_2_ intensity index (CI) of final energy consumption

To assesses energy efficiency and environmental sustainability of the energy system, the study employed the CI model to measure the carbon emission intensity of Ghana using CO_2_ data from the world Bank group^50^.

$$CI=\frac{{\mathop \sum \nolimits_{{i=1}}^{s} {\text{CO}}{_2}}}{{\mathop \sum \nolimits_{{i=1}}^{s} x }}$$

Where CO_2_ is kiloton of Carbon Dioxide equivalent and x ktoe of final energy consumed.

$${\text{Total~cx~for~}}2020 = 39070.29$$

$${\text{Total~ex~for~}}2020 = 8601$$

$$CI=\frac{{39070.29}}{{8601}}$$

$$CI=4.54$$

**Fig. 7:**
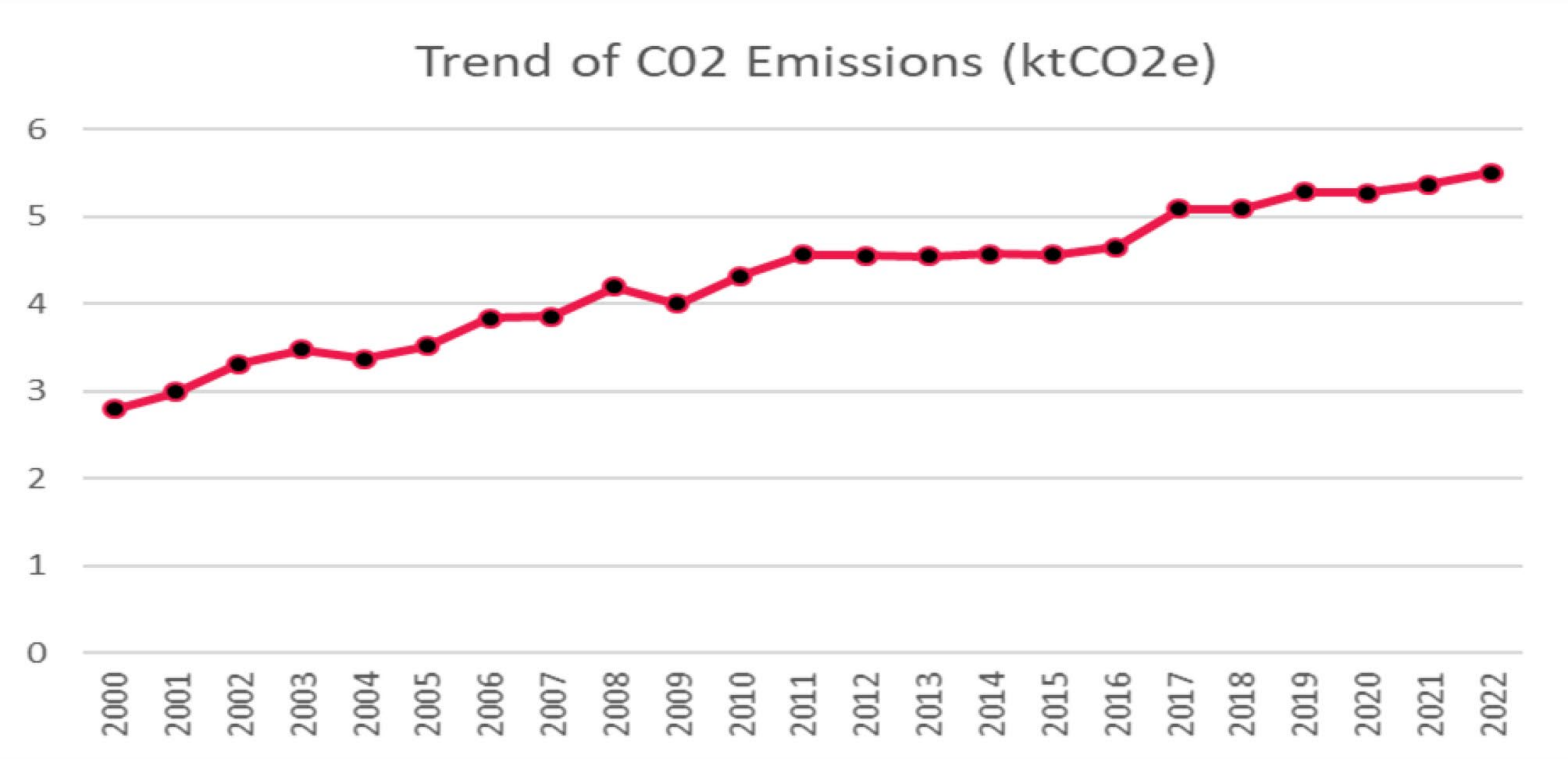
CO_2_ Intensity of Ghana. Source: Authors computations

Figure 7 presents a 23-year trend analysis of carbon intensity (ktCO2/ktoe) in Ghana:

Figure 7 above illustrates the twenty-year trend of carbon intensity (ktCO_2_/ktoe) in Ghana from 2000 to 2020. The overall trend shows a steady increase in CO₂ intensity, indicating that Ghana’s energy production has become more carbon-intensive over time. There are periods of fluctuation, notably around 2004 and 2010, where CO_2_ intensity temporarily declined before continuing its upward trajectory. The general rise suggests increased reliance on fossil fuels or less efficient energy production methods, contributing to higher emissions per unit of energy consumed. The trend underscores the need for cleaner energy transitions and efficiency improvements to mitigate carbon intensity and ensure environmental sustainability.

Ghana’s post-COVID recovery plan emphasized scaling up renewable energy, especially through mini-grid and off-grid solar projects targeting rural and peri-urban areas^51^. However, from the chart, there is no visible decline or flattening in the CO₂ emissions trend during the post-COVID years (2020–2022). Emissions continue to rise, even though at a slower pace compared to the pre-COVID years.

### Summary of indices analysis

Summary of 2022 indices results are shown in Table 7.

**Table 7:** Summary of indices. Source: Authors computations.

Type of index	Indicator	Number of variables	Index	Period	Interpretation
The Herfindahl-Hirschman Index (HHI_1_)	Market Concentration Levels	6	0.23	2022	Good
The Herfindahl-Hirschman Index (HHI_2_)	Power Sector Market Structure	14	0.09	2022	Good
The Shannon–Wiener Index (H)	Diversity and Dependence	6	1.51	2022	Good
The Simpson Index (D)	Diversity and Dependence	6	4.31	2022	Good
Adjusted Shannon–Wiener Newmann Index (SWNI_1_)	Political Risk	4	0.37	2022	SWNI_1_ lowers the level of H
Adjusted Shannon–Wiener Newmann Index (SWNI_2_)	Indigenous Production	4	0.98	2022	SWNI_2_ raises the level of SWNI_1_
Carbon Intensity (ktCO2/ktoe)	Emission levels	3	5.50	2022	High level of emissions

## Analysis and discussions

An HHI_1_ of 0.23 indicates a relatively low concentration and a diverse energy market, which can be seen as a positive factor for the energy diversity profile of Ghana and enhances energy security. The trend of the HHI_1_ from the past ten years shows great improvement in terms of energy source diversity for the country. Within the 10-year period, the HHI_1_ showed continuous decline from 2016 index of 0.287 which is the second higher index to only the 2013 index of 0.291. After 2016 the index declines over the years to 0.232 in 2022, the lowest within the 10-year period.

The test for the power sector market structure resulted in an HHI_2_ of 0.09 which is quite good for the energy market structure. It suggests that the energy sector is characterized by a significant number of energy companies with relatively balanced market shares. A signal that the energy industry is promoting competition and reducing dependency on a few dominant energy sources. This level of competition could help reduce energy prices and increase energy as to the poor. Healthy competition could also contribute to energy security by reducing the country’s vulnerability to disruptions in a single energy source or company.

The HHI_1&2_, however, has its own shortcomings as it fails to consider domestic production and political risk into consideration and therefore may not be seen as a comprehensive analysis of the energy security dimensions of a country. Even though, it is a precise metric, it is important to compare HHI with other indices when analysing energy security issues, especially for countries that import a considerable portion of their energy needs.

An assessment of the Shannon–Wiener Index (H) for the 2022 energy dataset of Ghana is 1.5. The higher H indicates a more competitive market. The Shannon–Wiener Index indicates the diversity of the energy portfolio of Ghana which suggests good levels of the energy mix indicating greater diversity. The energy is being generated from a wide variety of sources with relatively balanced contributions. This is a positive sign for energy security, as it implies that the country is not heavily dependent on a single energy source. The modelling also tracked the Shannon–Wiener Index for Ghana energy profile for the last ten years as illustrated in Fig. 4 to make inferences about changes in the energy diversity within Ghana’s energy sector. The H trend just as the HHI_1_ trend shows that the energy diversity greatly improved from 2016 which hard the second lowest H of 1.36 and 2022 showing the highest H of 1.5. Among the reasons for the low H in 2016 was due to the energy crisis that hit Ghana from 2013 and heightened at 2016. Many interdependent power producers were brought in to help improve the energy diversity thereby lowering the H continuously afterwards.

Just as the HHI, the main limitations remain. The Shannon–Wiener Index also cannot take domestic production separately from the imports, and the political risk cannot be incorporated into the model.

To obtain a wide perspective on the energy variety and dependence and to validate the HHI and H, the study applied the Simpson Index model with the same dataset. We computed a reverse Simpson Index value of 4.3 which suggest a relatively diversified energy mix. This diversification is positive with regard to the energy security of the country, as it indicates that the country is not heavily reliant on a single or a few dominant energy sources.

The SWNI_1_ measures the energy security of external energy supply in Ghana. It assesses the proportion of energy imported and the political risk associated with the origin of the imports. The HHI_1_ value of 0.23 suggests moderate concentration, indicating Ghana has some level of diversification. However, this does not account for geopolitical risk. In contrast, the SWNI_1_ index, which integrates political stability of import origin, reveals heightened vulnerability due to reliance on politically unstable regions for energy imports. This distinction highlights the importance of using multi-dimensional indices to assess different aspects of energy security. Thus, the SWNI_1_ makes much sense when compared to the H index. SWNI_1_ of 0.48 is less than H of 1.51. 0.48 < 1.51 shows a decrease in the energy profile situation because the ASWI_1_ incorporated energy imports sources. An indication that most of the origin of Ghana’s energy imports do not have strong political stability factors and therefore, imports from such unstable regions of the world tend to reduce the original Shannon–Wiener Index drastically, thereby making the country vulnerable to supply disruptions. This revelation borders on the energy security of the country as it introduces uncertainty on the supply chain of energy imports. The consequences of such uncertainties depend on how much energy the economy relies on through imports versus domestic production. According to the Energy Commission (2023), about 28% of the energy supplied (primary energy supplied plus petroleum products) for 2022 was imported^52^. Ghana imports a significant portion of its Natural Gas, crude oil and refined petroleum from politically unstable regions, particularly Nigeria, the United States and United Kingdom^53–55^. Nigeria is a key supplier of natural gas through the West African Gas Pipeline (WAGP). However, the country frequently experiences political unrest, insurgency (e.g., Niger Delta militant attacks), and oil theft, which disrupts supply. When supply is interrupted, Ghana is forced to buy more expensive alternatives, leading to higher electricity tariffs and fuel prices. For example, during the 2013–2015 power crisis, disruptions in Nigerian gas supply forced Ghana to rely on more expensive crude oil for power generation, worsening load shedding (‘dumsor’) and economic slowdown^56^. The United States and United Kingdom are major suppliers of crude and refined petroleum products and have geopolitical tensions, trade policies, and fluctuations in crude oil production that can lead to fuel price volatility in Ghana^57,58^. The impact on the ordinary Ghanaian is higher fuel prices and increase transportation costs, leading to rising food prices and inflation. Higher household energy bills make basic electricity and cooking fuel less affordable. Businesses, particularly transport companies and manufacturers, pass on the increased costs to consumers, further straining household budgets.

By introducing indigenous energy production as an additional factor that represents the share of energy sources produced domestically to the Adjusted Shannon Wiener Index (SWNI_2_) the SWNI_2_ increased to 0.98 from SWNI_1_ of 0.48. A SWNI_2_ of 0.98 > 0.37 which indicate domestic production can increase the energy source diversity and enhance the energy supply chain positively thereby enhancing the energy security situation. The introduction of indigenous energy production has significantly changed our Shannon–Wiener Index results and presenting a different perspective of the energy security analysis.

Ghana’s energy security profile within the West African sub-region is essential. Nigeria, although a major oil and gas producer, faces persistent energy insecurity due to low refining capacity, high dependency on fuel imports, and recurring gas pipeline disruptions. This overreliance on imported refined products introduces significant geopolitical and infrastructural vulnerabilities. In contrast, Côte d’Ivoire has enhanced energy stability through greater reliance on hydropower and integration into the West African Power Pool (WAPP), enabling cross-border electricity trade. Ghana’s performance sits between these two extremes more diversified than Nigeria, yet still constrained by external supply dependencies and modest renewable integration. The contrasts highlight the varied trajectories of energy transition and resilience across West Africa.

Carbon Intensity of 5.50 kt of CO_2_ per ktoe of energy consumed for 2022 is relatively high in the context of carbon emissions associated with energy consumption. This means that for every kiloton of oil equivalent (ktoe) of energy consumed, approximately 5.50 kilotons (or 5498.45 metric tons) of carbon dioxide (CO_2_) are emitted into the atmosphere. This is not a good indicator for the energy security context of the country as these emission levels have implication for climate change. It is no surprise that Ghana is among the top ten CO₂ emitters in Africa, according to the IEA^59^. Even though, Ghana’s emissions are relatively moderate, compared to countries like South Africa, Egypt, Algeria and Nigeria that tops the list, Carbon Intensity of 5.50 kt is a concern for Ghana in terms of meeting environmental sustainability goals such as the NDCs and United Nations Sustainable Development Goals (SDGs). It is therefore, critical for Ghana to expand renewable energy investments, particularly in solar, wind, and hydro, to reduce dependence on fossil fuels. Other measures that can help the reduction of Ghana’s CO_2_ emissions are carbon Pricing and Regulation, and promotion of electric vehicles (EVs) and public transportation. Also, Encouraging energy efficiency programs in industries and households could lower emissions further. As part of its post-COVID recovery strategy, Ghana placed a strong emphasis on accelerating the adoption of renewable energy, with a particular focus on expanding mini-grid and off-grid solar energy systems. These initiatives were primarily aimed at increasing energy access in rural and peri-urban communities, reducing dependence on fossil fuels, and promoting sustainable development.

Despite these efforts, the CO₂ emissions trend from 2020 to 2022 does not show a clear decline or stabilization. The data indicate that emissions have continued to rise during the post-COVID period, although the rate of increase appears to have moderated slightly compared to the sharper upward trend observed in the years leading up to the pandemic. This suggests that while renewable energy projects are being implemented, their scale and impact may not yet be sufficient to offset the broader national emissions growth driven by increasing energy demand and continued reliance on conventional energy sources.

## Conclusion and policy implications

HHI_1_ score of 0.23 and H index of 1.5 as revealed by the results, reflects a moderately concentrated energy market in Ghana. This finding underscores the importance of diversifying energy sources and enhancing energy resilience to mitigate potential vulnerabilities. The HHI_2_ of 0.09 revealed a very competitive market structure for the power generation sector which is a positive indicator for energy systems of the country. However, the share of renewables such as wind and solar in the power generation mix is negligible and raising environmental concerns.

Ghana, like many other nations, faces energy security challenges influenced by various factors such as supply disruptions, market concentration, and external shocks. The existing energy system seems to have addressed these challenges in the short to medium- term. However, our imports policy must ensure that the origin of imports have high political stability factors with long term supply contracts. Also, fostering international collaborations and trade partnerships can improving energy security.

Government must also take proactive steps to ensure a robust and sustainable energy future for the nation as a Carbon Intensity of 5.50 kt of CO_2_ per ktoe is relatively high emissions. The continued rise in CO₂ emissions in Ghana even during the post-COVID period, despite the government’s increased investment in renewable energy, signals a need for more comprehensive and aggressive climate and energy policies. While the expansion of mini-grid and off-grid solar projects is a positive step toward decarbonizing the energy sector, the current scale and speed of implementation appear insufficient to significantly influence national emission levels in the short term. This indicates that Ghana’s renewable energy interventions must be better integrated into a broader national strategy that includes phasing out fossil fuel-based generation especially in the transport sector that consumes about 33% of Ghana energy. Other interventions should be targeted at improving energy efficiency, and enforcing stricter emissions regulations. Specific policies such as incentives for solar and wind energy, improvements in grid reliability, and regional energy trade agreements could enhance long-term security while reducing environmental impact.

The energy security analysis of Ghana has provided valuable insights into the country’s energy landscape. The study provides a comprehensive assessment of Ghana’s energy security by employing a multi-indices approach, incorporating the HHI, H Index, Simpson Index, SWNI and CI to assess Ghana’s energy sources and environmental sustainability. Furthermore, this study’s multi-indices approach offers a more nuanced understanding of Ghana’s energy security compared to previous studies that often rely on single-index evaluations. By integrating multiple metrics, we capture the interplay between diversity, reliability, and sustainability, providing a more holistic assessment of vulnerabilities and strengths in the energy sector.

This study serves as a foundation for future studies for researchers interested in the intersection of energy security and environmental sustainability, particularly in the context of emerging economies like Ghana. It emphasises the urgency of taking proactive measures to ensure a secure and sustainable energy landscape for countries and regions. Ultimately, achieving energy security is essential not only for the well-being of Ghanaians but provides a step towards achieving the United Nations SDGs and halting global climate change.

### Study limitations

Despite the comprehensive nature of this study, several limitations must be acknowledged. First, data availability remains a significant constraint, for instance, the reliance on publicly available sources may introduce inconsistencies in reporting and classification. Second, the indices used; such as the HHI and the H index assume that diversity alone enhances energy security, which may overlook other critical factors such as infrastructure resilience, technological progress, or dynamic policy responses. Finally, external factors such as policy shifts, global energy price fluctuations, and climate change impacts were not directly modelled but are important considerations for future research. Addressing these limitations through real-time data analytics and scenario-based modelling could enhance future assessments of Ghana’s energy security. Therefore, future research should incorporate scenario-based modeling, energy storage capacity, and grid flexibility metrics to better assess the evolving nature of energy systems.

## Data Availability

The datasets generated during and/or analysed during the current study are available from the corresponding author on reasonable request.
